# Risk factors of acute myocardial infarction in middle-aged and adolescent people (< 45 years) in Yantai

**DOI:** 10.1186/s12872-015-0102-5

**Published:** 2015-09-29

**Authors:** Hong Du, Chang-yan Dong, Qiao-yan Lin

**Affiliations:** Cardiology Ward, the Yantai Yuhuangding Hospital, No. 20, Yuhuangding Eastern Road, Yantai, 264000 Shandong Province China; Chinese and Western Medicine Ward, the Yantai Yuhuangding Hospital, No. 20, Yuhuangding Eastern Road, Yantai, 264000 Shandong Province China; Cardiology in Intensive Care Unit, the Yantai Yuhuangding Hospital, No. 20, Yuhuangding Road, Yantai, 264000 Shandong Province China

**Keywords:** Risk factors, Acute myocardial infarction, Middle-aged and adolescent people, Developed medium-sized coastal city, China

## Abstract

**Background:**

Yantai is a developed medium-sized coastal city in Eastern China, having a population of 1.6845 million. With the development of economy, some middle-aged and adolescent people (< 45 years) devote themselves to work and suffer from greater stress, which makes them ignore their own health. Moreover, they have unhealthy lifestyles and lack the knowledge of cardiovascular risk factors.

**Objectives:**

To identify the risk factors for first acute myocardial infarction in middle-aged and adolescent people in Yantai, a developed medium-sized coastal city in Eastern China.

**Methods:**

A total of 154 consecutive patients with first acute myocardial infarction (< 45 years), were enrolled in case group, and 462 patients without myocardial infarction were enrolled in control group. Three controls with the same sex and age were matched to each case. The risk factors were identified with univariate and multivariate analysis.

**Results:**

Unhealthy food habit (eating seafood and meanwhile drinking beer), hypertension, current smokers, self-perceived stress, diabetes mellitus, obesity, sleep insufficience, hypercholesterolaemia and fatigue were independent risk factors for first acute myocardial infarction (*P* < 0.05).

**Conclusions:**

Besides those recognized risk factors for cardiovascular disease (hypertension, hypercholesterolemia, diabetes mellitus and smoking), eating seafood and meanwhile drinking beer, self-perceived stress, sleep insufficience, obesity and fatigue were also the risk factors for first acute myocardial infarction in middle-aged and adolescent people in Yantai.

## Background

As a leading cause of death in developed countries [1], cardiovascular disease (CVD) has an elevating incidence and prevalence in developing countries in recent years [2], which may be caused by urbanization, adoption of western life styles and aging of population. China has the biggest CVD burden as the biggest developing country [3]. In 2010, Chinese population with an age greater than 40 years has about 8 million of myocardial infarction (MI) patients, and the number will increase persistently in the next two decades. Moreover, the age of onset is younger for first acute myocardial infarction (AMI) in Chinese population [4]. However, younger patients with first AMI have rarely been researched.

Yantai is a developed medium-sized coastal city in Eastern China, having a population of 1.6845 million. With the development of economy, some middle-aged and adolescent people (< 45 years) devote themselves to work and suffer from greater stress, which makes them ignore their own health. Moreover, they have unhealthy lifestyles and lack the knowledge of cardiovascular risk factors (CVRF). Especially, many middle-aged and adolescent people like to eat seafood and meanwhile drink beer with the improvement of living standard in Yantai, which was uncommon in the before. Besides, we observed that the number of middle-aged and adolescent patients with primary AMI had an annual increase in our hospital in recent years. If the risk factors for first AMI could be identified in middle-aged and adolescent people, early prevention would be able to be administered.

The objective of the paper was to identify the risk factors for first AMI in middle-aged and adolescent people in Yantai, and then provide useful information for the prevention of primary AMI among middle-aged and adolescent people in the cities similar to Yantai.

## Methods

### Participants

A total of 154 consecutive patients with first AMI (< 45 years), including 127 males and 27 females, were enrolled in case group in the Yantai Yuhuangding Hospital from June 2005 to June 2013. A total of 462 patients without MI were enrolled in control group, including 381 males and 81 females. Three controls with the same sex and age were matched to each case. All the participants provided written informed consent, and the study was approved the Ethical Committee of the Yantai Yuhuangding Hospital.

### Diagnostic criteria

1. Cardiac biomarkers had a rise (to at least one time of the upper normal value) or fall after rise (to at least one time of the upper normal value). 2. At least one of the following characteristics presented: [1] The patient had clinical symptoms of myocardial ischemia. [2] ECG showed new ST segment change or left bundle-branch block. [3] ECG showed pathological Q wave. [4] Imaging evidence showed new loss of myocardial activity or regional wall motion abnormality.

### Variables and measurement

The selected factors in the study were as follows: self-perceived stress, physical activity, sleep, fatigue, smoking status, alcohol consumption, food habit, family history of coronary heart disease (CHD), body mass index (BMI), occupation, family income, education, blood pressure, blood glucose, and cholesterol. The information about the selected factors was collected by interview, physical examinations and referring to medical records.

Self-perceived stress was defined as yes or no according to the question “how many days have you felt stressful or depressive in a week?” Yes was determined if participants have felt stressful or depressive on three or more days, and no was determined if participants have felt stressful or depressive on two or less days. Physical activity was defined as active or inactive according to the question “have you had an activity of 30 min on five or more days in a week?” Sleep was defined as sufficient or insufficient according to the question “how many days have you had at least six hours of sleep duration in a week?” Sufficient was determined if participants had at least six hours of sleep duration on five or more days, and insufficient was determined if participants had at least six hours of sleep duration on four or less days. Fatigue was defined as yes or no according to the question “have you experienced fatigue on three or more days in a week?” Smoking status was defined as current smokers, ex-smokers or non-smokers according to the question “how many cigarettes have you smoked per day?” The people that answered at least one cigarette per day were defined as current smokers, and the people that answered at least one year of smoking cessation was defined as ex-smokers. Alcohol consumption was defined as yes or no according to the question “have you drunk at least 100 g of distillate spirit or 500 g of beer on three or more days in a week?” Food habits were defined as unfavorable or favorable according to the question “how many times have you eaten seafood and meanwhile drunk beer in a meal in a week?” Unfavorable was determined if participants had at least two times of eating seafood and meanwhile drinking beer in a meal, and favorable was determined if participants had at most once. Family history of CHD was defined as yes or no according to the question “have you had one more parent or sibling with diagnosed CHD?” BMI was calculated according to the formula that weight (kg) was divided by squared height (m^2^). BMI (18.5-24.9 kg/m^2^) was defined as normal, (25–29 kg/m^2^) was defined as overweight, and (≥30 kg/m^2^) was defined as obesity. Occupation was defined as physical work or intellectual work. According to family income, all the participants were categorized into three groups: low income group (< 10,000 RMB/year), middle income group (10,000–20,000 RMB/year) and high income group (> 20,000 RMB/year). According to the time of education completed, all the participants were categorized into three groups: primary (1–8 years), secondary (9–12 years), and postsecondary (≥13 years).

Hypertension was determined if blood pressure was ≥140/90 mmHg [5] and/or the patient was receiving antihypertensive drugs. Diabetes mellitus was determined if fasting plasma glucose was ≥7.0 mmol/L or 2 h oral glucose tolerance test (OGTT) glucose was ≥11.1 mmol/L [6] and/or the patient was receiving antidiabetic drugs. Hypercholesterolaemia was determined if total serum cholesterol was ≥ 5.16 mmol/L (200 mg/dl) [7] and/or the patient was receiving cholesterol lowering treatment.

### Statistical analysis

All the statistical analyses were carried out with the SPSS version 17.0 for Windows (SPSS Inc., USA). The quantitative variables were expressed as mean ± SD, and the qualitative variables were expressed as percentage. The quantitative variables were analyzed with Student's *t* test. The qualitative variables were analyzed with chi-square test or Fisher exact test. The variables with a *P* value less than 0.10 in univariate analysis were included in the multivariate analysis with a backward stepwise logistic regression model. Multivariate logistic regression analyses were then performed to determine the risk factors correlated with the death of the elderly patients with AOSC. Significance was set at *P* < 0.05.

## Results and discussion

The average age was 36.8 ± 5.2 years for the cases, and men accounted for 82.5 %. The difference was not significant in the age of first onset between men and women (36.5 ± 5.1vs37.7 ± 5.5 years, *P* > 0.05). Coronary catheterization was performed in all the cases.

According to the results of univariate analysis, the variables correlated with first AMI were as follows: self-perceived stress, sleep, fatigue, smoking status, food habit, BMI, blood pressure, blood glucose and cholesterol (Table 1). Multivariate analysis showed that unhealthy food habit (eating seafood and meanwhile drinking beer), hypertension, current smokers, self-perceived stress, diabetes mellitus, obesity, sleep insufficience, hypercholesterolaemia and fatigue were independent risk factors for first AMI (Table 2). The OR ranged from 1.70 to 3.18 among these risk factors, and the OR was highest for unhealthy food habit and lowest for fatigue (Table 2).

**Table 1:** Results of univariate analysis of the risk factors for first AMI

Variables	AMI	Non-AMI	*χ* ^2^/*t*	*P*
Self-perceived stress (yes)	36	60	9.477	0.002
Physical inactivity	36	79	2.997	0.083
Sleep insufficience	52	100	9.13	0.003
Fatigue (yes)	40	79	5.863	0.016
Current smokers	95	193	18.495	<0.001
Alcohol consumption (yes)	61	150	2.617	0.106
Eating seafood and meanwhile drinking beer	71	98	35.947	<0.001
Family history of CHD	19	46	0.694	0.405
Obesity	42	89	11.064	0.004
Physical work	67	208	0.107	0.743
Low family income	23	84	2.586	0.274
Education (none)	43	98	3.758	0.153
Hypertension	49	86	11.766	0.001
Diabetes mellitus	23	34	7.894	0.005
Hypercholesterolaemia	42	83	6.186	0.013

**Table 2:** Results of multivariate analysis of the risk factors for first AMI

Risk factors	Wald	*P* value	OR	95 % CI
Eating seafood and meanwhile drinking beer	20.537	<0.001	3.18	1.647-4.893
Hypertension	8.026	0.009	2.42	1.315-3.652
smoking status (compared with non-smokers)				
Current smokers	9.875	0.001	2.36	1.429-3.477
ex-smokers	2.238	0.073	1.21	0.736-1.704
Self-perceived stress	6.182	0.013	2.29	1.302-3.315
Diabetes mellitus	5.329	0.018	2.21	1.211-3.289
BMI (compared with normal)				
Obesity	4.251	0.024	2.04	1.192-3.024
Overweight	1.404	0.142	1.54	0.891-2.213
Sleep insufficience	4.832	0.021	1.85	1.105-2.758
Hypercholesterolaemia	3.006	0.041	1.71	1.048-2.396
Fatigue	2.817	0.043	1.70	1.034-2.282
Physical inactivity	1.327	0.159	1.50	0.863-2.206

As a coastal city in Eastern China, Yantai had a rapid development of economy in the past two decades, which led to dietary and lifestyle changes [8], especially in middle-aged and adolescent people. These changes could increase the risk for CVD. In our study, other factors were associated with first AMI in middle-aged and adolescent people in Yantai, except for physical activity, alcohol consumption, family history of CHD, occupation, family income and education.

We found that eating seafood and meanwhile drinking beer was positively associated with first AMI. Firstly, consumption of seafood can increase the prevalence of hyperuricemia, which possibly results from the high content of purine in seafood [9]. Secondly, a recent study shows that heavy alcohol consumption of wine can increase the prevalence of hyperuricemia [10], and another study shows that alcohol consumption is directly associated with hyperuricemia [11]. Thirdly, drinking beer and spirits can increase the risk for gout, whereas a moderate consumption of wine cannot [12]. Eventually, hyperuricemia is associated with severity of coronary artery disease (CAD) [13]. However, our study showed that alcohol consumption was not associated with first AMI. The possible reason is that moderate alcohol consumption can reduce the risk for CVD [14] and people tend to drink heavily when they eat seafood.

It is reported that hypertension [15-17], hypercholesterolemia [18], diabetes mellitus [17, 19] and smoking [17] are the risk factors for CVD. Our study showed that hypertension, current smoking, diabetes mellitus and hypercholesterolemia were the risk factors for first AMI in middle-aged and adolescent people in Yantai. Moreover, we found that smoking cessation could reduce the risk for first AMI compared with current smoking, but the result needed to be further confirmed by a larger sample.

A study shows that work stress is a risk factors for CVD [20], and another study shows that stress is directly associated with coronary heart disease [21].We found that self-perceived stress was positively associated with first AMI. However, some studies show that stress is not associated with CVD [22-24]. We found that sleep insufficience was associated with first AMI, which was consistent with the previous study [25]. We also found that obesity and fatigue were associated with first AMI, but overweight was not.

The limitations of the study included: [1] The participants were chose from a small population (inpatients); [2] The relationship was not studied between HIV infection and first AMI because only 5 participants had HIV infection among all the participants; [3] The risk factors of first AMI were not studied in the population > 45 years.

## Conclusions

In conclusion, besides those recognized risk factors for CVD (hypertension, hypercholesterolemia, diabetes mellitus and smoking), eating seafood and meanwhile drinking beer, self-perceived stress, sleep insufficience, obesity and fatigue were also the risk factors for first AMI in middle-aged and adolescent people (< 45 years) in Yantai. In a next study, the risk factors of first AMI will be comprehensively studied for inhabitants in Yantai, and a prospective cohort study will be performed, aiming to assess the outcome associated with these emerging risk factors.
